# MALDI-TOF Mass Spectrometry for the Detection and Differentiation of *Entamoeba histolytica* and *Entamoeba dispar*

**DOI:** 10.1371/journal.pone.0122448

**Published:** 2015-04-15

**Authors:** Adriana Calderaro, Maddalena Piergianni, Mirko Buttrini, Sara Montecchini, Giovanna Piccolo, Chiara Gorrini, Sabina Rossi, Carlo Chezzi, Maria Cristina Arcangeletti, Maria Cristina Medici, Flora De Conto

**Affiliations:** Unit of Microbiology and Virology, Department of Clinical and Experimental Medicine, University of Parma, Parma, Italy; Centro de Investigacion y de Estudios Avanzados del Instituto Politecnico Nacional, MEXICO

## Abstract

Detection of *Entamoeba histolytica* and its differentiation from *Entamoeba dispar* is an important goal of the clinical parasitology laboratory. The aim of this study was the identification and differentiation of *E*. *histolytica* and *E*. *dispar* by MALDI-TOF MS, in order to evaluate the application of this technique in routine diagnostic practice. MALDI-TOF MS was applied to 3 amebic reference strains and to 14 strains isolated from feces that had been differentiated by molecular methods in our laboratory. Protein extracts from cultures of these strains (axenic cultures for the 3 reference strains and monoxenic cultures for the 14 field isolates) were analyzed by MALDI-TOF MS and the spectra obtained were analyzed by statistical software. Five peaks discriminating between *E*. *histolytica* and *E*. *dispar* reference strains were found by protein profile analysis: 2 peaks (8,246 and 8,303 Da) specific for *E*. *histolytica* and 3 (4,714; 5,541; 8,207 Da) for *E*. *dispar*. All clinical isolates except one showed the discriminating peaks expected for the appropriate species. For 2 fecal samples from which 2 strains (1 *E*. *histolytica* and 1 *E*. *dispar*) out of the 14 included in this study were isolated, the same discriminating peaks found in the corresponding isolated amebic strains were detected after only 12h (*E*. *histolytica*) and 24h (*E*. *dispar*) of incubation of the fecal samples in Robinson’s medium without serum. Our study shows that MALDI-TOF MS can be used to discriminate between *E*. *histolytica* and *E*. *dispar* using *in vitro* xenic cultures and it also could have potential for the detection of these species in clinical samples.

## Introduction

Matrix-assisted laser desorption/ionization–time of flight mass spectrometry (MALDI-TOF MS) has recently revolutionized clinical microbiology [1], proving to be a reliable approach for solving certain problems linked to the identification and strain differentiation of microorganisms, and overcoming limitations of conventional methods [2]. This technique allows the rapid, accurate, and inexpensive identification of microorganisms from both cultures and biological samples. It is now also used in diagnostic microbiology laboratories where it has emerged as a first-line method for the accurate identification of bacteria, but few data are available for protozoa [3]. As far as human intestinal parasites as concerned, the application of MALDI-TOF MS has been limited to obtaining general parasitic proteome data [4,5], to characterizing specific biomarkers for discriminating between *Cryptosporidium* and *Giardia* species [6,7], and to determining the subtype of *Blastocystis* sp. isolates from liquid xenic cultures [1].

Detection of *Entamoeba histolytica*, the causative agent of amebiasis, and its differentiation from *Entamoeba dispar* is an important goal of the clinical parasitology laboratory [8, 9]. Amebiasis is one of the most common causes of death from protozoan parasitic diseases, second only to malaria, with approximately 50 million cases and 100,000 deaths annually, as reported by the WHO [8–10]. Some *E*. *histolytica* infections cause dysentery and diarrhea while a few progress to the development of extra-intestinal complications such as liver abscess [11]. The existence of two genetically distinct but morphologically identical species, i.e. *E*. *histolytica* and *E*. *dispar* [12], has been confirmed through extensive genetic, immunological, biochemical and biomolecular analysis [10,13]. The identification of *E*. *dispar* as a separate but non-pathogenic species that does not require treatment has highlighted the need for alternative and improved detection methods [8] able to differentiate between the two microorganisms [14].

In the 2002 Blessmann et al. [15] developed a Real-time PCR assay for the detection and differentiation of *E*. *histolytica* and *E*. *dispar* in fecal samples. In our laboratory we demonstrated the utility of this Real-time PCR assay for the identification and differentiation of *E*. *histolytica* and *E*. *dispar* in clinical samples to routinely diagnose amebiasis [9]. Molecular methods overcome the limitations of microscopy and culture, which are able to detect but do not identify and differentiate the two species [8,10,16] but are cumbersome and expensive [9,17].

The aim of this study was the identification and differentiation of *E*. *histolytica* and *E*. *dispar* by MALDI-TOF MS analysis, using parasites grown both axenically and in xenic cultures from clinical samples in order to further evaluate the application of this approach in diagnostic practice.

## Results

**Fig 1:**
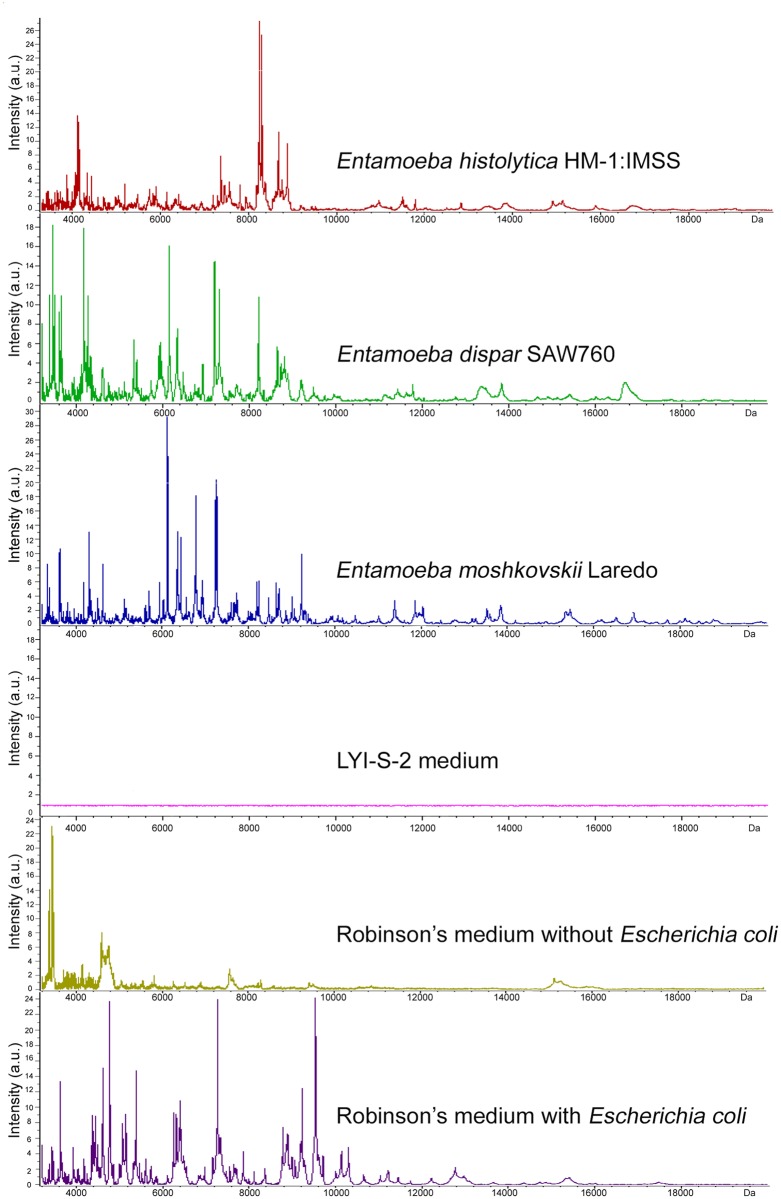
Average spectra obtained by MALDI-TOF MS analysis. Average spectra obtained for the replicates acquired by MALDI-TOF MS of the 3 *Entamoeba* sp. reference strains (*E*. *histolytica* HM-1:IMSS, *E*. *dispar* SAW760 and *E*. *moshkovskii* Laredo) and the 3 different media (LYI-S-2, Robinson’s medium with and without *Escherichia coli*) analyzed in this study.

Each of the three *Entamoeba* reference strains (*E*. *histolytica* HM-1:IMSS, *E*. *dispar* SAW760, and *E*. *moshkovskii* Laredo) yielded a proteic profile that was reproducible in all the conditions tested; neither inter-assay nor intra-assay variability were observed and no peaks were found for LYI-S-2 axenic medium (Fig. 1). In Fig. 1, the spectra obtained for Robinson’s medium with and without *Escherichia coli* are also shown.

Analysis of the spectra was performed by selecting a range of molecular weight between 4,500–10,000 Da, which includes the major differences between the proteic profiles of the different strains. Between 10,000 and 20,000 Da peaks were not found and between 2,000 and 4,500 Da the spectra were not considered discriminating.

**Fig 2:**
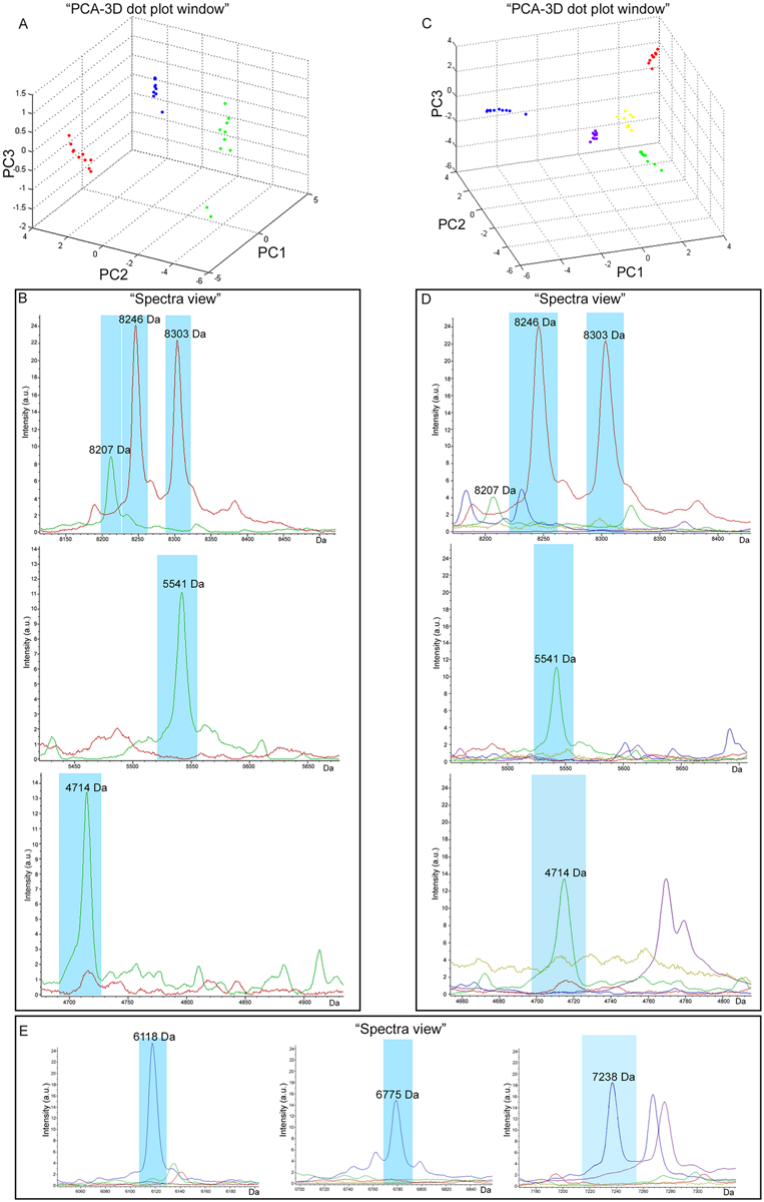
Comparative analysis of the spectra of the reference strains *E*. *histolytica* HM-1:IMSS, *E*. *dispar* SAW760 and *E*. *moshkovskii* Laredo. **(A)** “PCA-3D dot plot windows” showing 3 separate clusters in the spectra of the 3 different reference strains. **(B)** “Spectra view” of the 5 discriminating peaks in the *E*. *histolytica* and *E*. *dispar* average spectra. **(C)** “PCA-3D dot plot windows” showing 5 separate clusters in the spectra of the 3 different reference strains in Robinson’s medium with and without *Escherichia coli*. **(D)** “Spectra view” of the 5 discriminating peaks of *E*. *histolytica* and *E*. *dispar* average spectra compared with those of *E*. *moshkovskii* and Robinson’s medium with and without *Escherichia coli*. **(E)** “Spectra view” of the 3 discriminating peaks of *E*. *moshkovskii* average spectra compared with those of *E*. *histolytica*, *E*. *dispar*, and Robinson’s medium with and without *Escherichia coli*.

In Fig. 2A, replicates of the spectra of *E*. *histolytica* HM-1:IMSS and *E*. *dispar* SAW760 in separate clusters are shown and both are distinct from the cluster obtained for *E*. *moshkovskii* Laredo.

Data regarding *E*. *histolytica* HM-1:IMSS are reported in red, *E*. *dispar* SAW760 in green, *E*. *moshkovskii* Laredo in blue, and Robinson’s medium with and without *Escherichia coli* in violet and yellow, respectively.

Five peaks discriminating between *E*. *histolytica* and *E*. *dispar* strains were found: 2 peaks (8,246 and 8,303 Da) only in the *E*. *histolytica* profile and 3 (4,714 Da; 5,541 Da; 8,207 Da) only in the *E*. *dispar* profile (“spectra view” Fig. 2B; Table 1).

**Table 1:** Patterns of differentiating peaks of the 3 *Entamoeba* spp. reference strains (*E*. *histolytica* HM-1:IMSS, *E*. *dispar* SAW760 and *E*. *moshkovskii* Laredo) and MALDI-TOF MS results of *Entamoeba* spp. clinical isolates analyzed in this study in comparison with those obtained with Real-time PCR.

Reference Strains	Real-time PCR^a^	Peak mass (m/z) representing the protein size in Dalton^b^								MALDI-TOF MS
		**4,714** p = 0.0005	**5,541** p = 0.0001	**6,118** p< 0.00001	**6,775** p< 0.00001	**7,238** p< 0.00001	**8,207** p = 0.013	**8,246** p<0.000001	**8,303** p<0.000001	
*E*. *histolytica* HM-1:IMSS	*E*. *histolytica*	**-**	**-**	**-**	**-**	**-**	**-**	**+**	**+**	*E*. *histolytica*
*E*. *dispar* SAW760	*E*. *dispar*	**+**	**+**	**-**	**-**	**-**	**+**	**-**	**-**	*E*. *dispar*
*E*. *moshkovskii* Laredo	Negative	**-**	**-**	**+**	**+**	**+**	**-**	**-**	**-**	*E*. *moshkovskii*
**Clinical isolates**										
Strain 8026	*E*. *histolytica*	**-**	**-**	**-**	**-**	**-**	**-**	**+**	**+**	*E*. *histolytica*
Strain 373	*E*. *histolytica*	**-**	**-**	**-**	**-**	**-**	**-**	**+**	**+**	*E*. *histolytica*
Strain 369	*E*. *histolytica*	**-**	**-**	**-**	**-**	**-**	**-**	**+**	**+**	*E*. *histolytica*
Strain 1238	*E*. *histolytica*	**-**	**-**	**-**	**-**	**-**	**-**	**+**	**+**	*E*. *histolytica*
Strain 3291	*E*. *histolytica*	**-**	**-**	**-**	**-**	**-**	**-**	**+**	**+**	*E*. *histolytica*
Strain 1656	*E*. *histolytica*	-	+	-	-	-	+	-	-	^
Strain 1557	*E*. *dispar*	**+**	**+**	**-**	**-**	**-**	**+**	**-**	-	*E*. *dispar*
Strain 217	*E*. *dispar*	**+**	**+**	**-**	**-**	**-**	**+**	**-**	-	*E*. *dispar*
Strain 240	*E*. *dispar*	**+**	**+**	**-**	**-**	**-**	**+**	**-**	-	*E*. *dispar*
Strain 368	*E*. *dispar*	**+**	**+**	**-**	**-**	**-**	**+**	**-**	-	*E*. *dispar*
Strain 417	*E*. *dispar*	**+**	**+**	**-**	**-**	**-**	**+**	**-**	-	*E*. *dispar*
Strain 1110	*E*. *dispar*	**+**	**+**	**-**	**-**	**-**	**+**	**-**	-	*E*. *dispar*
Strain 2550	*E*. *dispar*	**+**	**+**	**-**	**-**	**-**	**+**	**-**	-	*E*. *dispar*
Strain 1382	*E*. *dispar*	**+**	**+**	**-**	**-**	**-**	**+**	**-**	-	*E*. *dispar*

^a^: 18S rDNA Real-time PCR able to differentiate between *E*. *histolytica* and *E*. *dispar* [9].

^b:^ p-value indicating the significance of each discriminating peak.

^-^: no peak found.

^+^: peak present.

^^^: Peaks not discriminating between *E*. *histolytica* and *E*. *dispar*.

For 4 discriminating peaks (4,714 Da; 5,541 Da; 8,246 Da; 8,303 Da) an Area Under Curve (AUC) value of 1 was obtained, while for the remaining peak (8,207 Da) an AUC value of 0.98 was obtained.

In order to verify the absence of interference between the 5 discriminating peaks of *E*. *histolytica* and *E*. *dispar* and the peaks from Robinson’s medium with and without *E*. *coli*, the “Principal Component Analysis (PCA)-3D dot plot window” was created (Fig. 2C).

The clusters belonging to Robinson’s medium with and without *E*. *coli* were separated both from one another and from the 3 reference strains. The peaks present in the spectra obtained for *E*. *moshkovskii* Laredo and Robinson’s medium with and without *E*. *coli* did not interfere with the discriminating peaks found for *E*. *histolytica* and *E*. *dispar* (“spectra view” Fig. 2D). Moreover, three peaks (6,118 Da; 6,775 Da; 7,238 Da) were found to be discriminant for *E*. *moshkovskii* Laredo (Fig. 2E) with an AUC value of 1.

The molecular weights of the 2 discriminating peaks of *E*. *histolytica* were found to correspond to only 2 specific proteins of *E*. *histolytica* present in GenBank: Amoebapore A (Accession number 1OF9_A; 77 amino acids; molecular weight 8,250 Da) and an unknown putative protein of *E*. *histolytica* HM-1:IMSS-A (Accession number ENY65454.1; 71 amino acids; molecular weight 8,300 Da). The *E*. *dispar* and *E*. *moshkovskii* specific peaks have no corresponding proteins in GenBank yet.

In order to assess whether the *E*. *histolytica* peaks observed in the MALDI-TOF MS spectra derived from axenically cultured parasites corresponded to these proteins, the *E*. *histolytica* protein pattern obtained after SDS-PAGE was analyzed. A large band was observed at the expected size.

**Fig 3:**
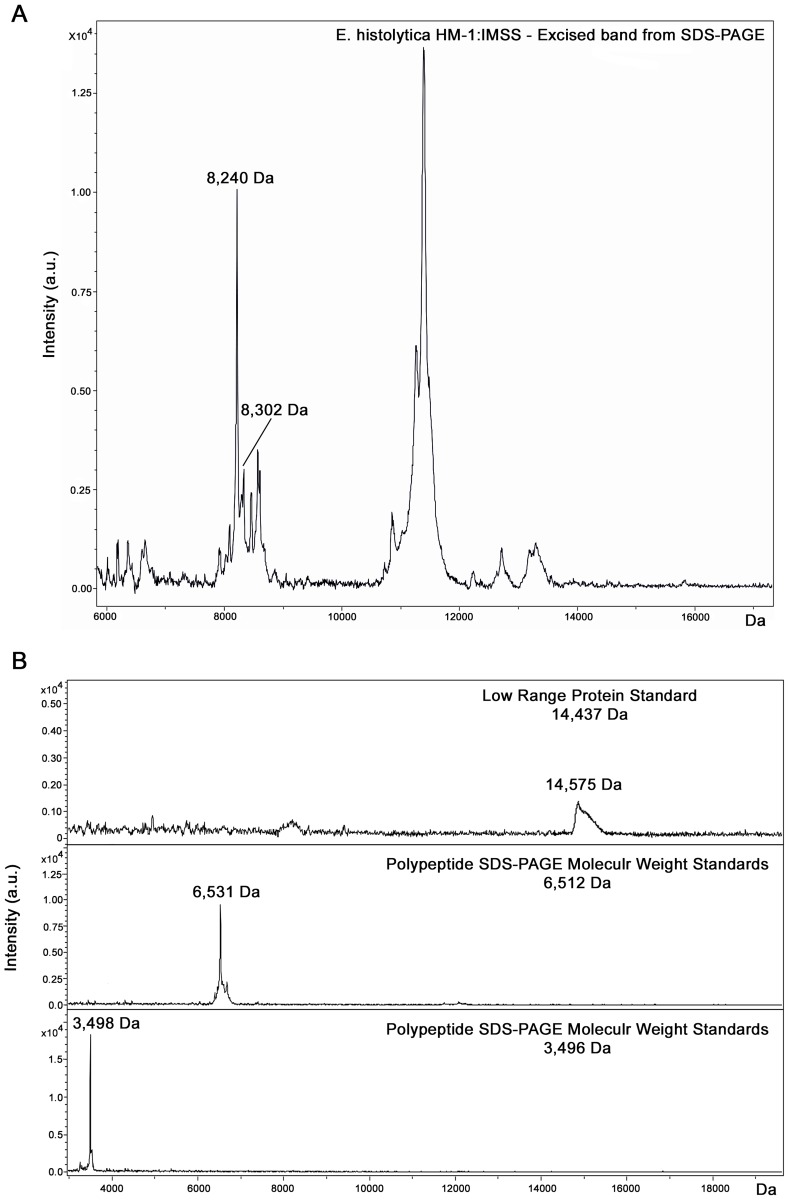
Spectra obtained by MALDI-TOF MS analysis after SDS-PAGE. **(A)** Mass spectrum of *E*. *histolytica* HM-1:IMSS excised protein gel band. **(B)** Protein mass spectra of molecular weight marker bands excised from the gel in the range of interest, corresponding to: insulin b chain, oxidized (3,496 Da), aprotinin (6,512 Da) and α-lactalbumin from bovine milk (14,437 Da) obtained by MALDI-TOF MS analysis.

The spectrum obtained by MALDI-TOF MS analysis of this band directly excised from Coomassie stained gel showed mass/charge ratios of 8,240 and 8,302 Da (Fig. 3A) which match with the 8,246 and 8,303 Da molecular weights of the *E*. *histolytica* discriminating peaks. This result was corroborated by the molecular weight marker bands in the range of interest (from 3,496 to 14,437 Da), excised and analyzed by MALDI-TOF MS, which showed a correspondence with the molecular weight obtained by SDS-PAGE (α-lactalbumin from bovine milk:14,575 Da; aprotinin: 6,531 Da; insulin beta chain oxidized: 3,498 Da) (Fig. 3B).

In order to verify the reliability of these discriminating peaks, 14 different clinical isolates belonging to the species *E*. *histolytica* (8026, 369, 373, 1238, 1656, 3291) and *E*. *dispar* (1557, 217, 240, 417, 368, 1110, 2550, 1382) were analyzed by MALDI-TOF MS (Table 1).

**Fig 4:**
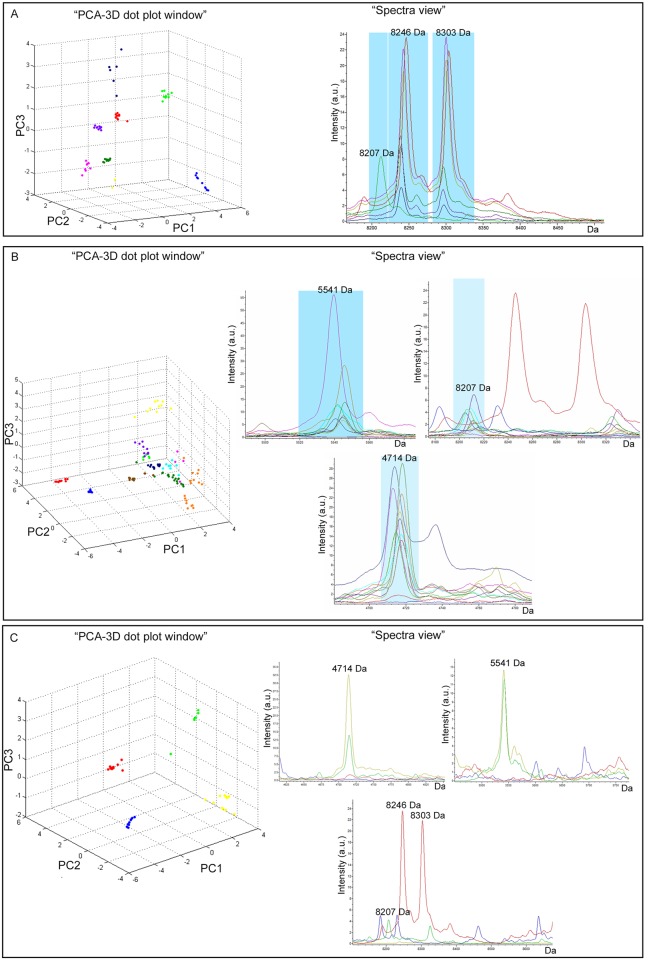
Comparative analysis of the average spectra obtained for clinical isolates and reference strains *E*. *histolytica* HM-1:IMSS, *E*. *dispar* SAW760, and *E*. *moshkovskii* Laredo spectra. **(A)** “PCA-3D dot plot windows” showing the spectra of *E*. *dispar* SAW760 (in green) and *E*. *moshkovskii* Laredo (blue) clustering separately from the group including *E*. *histolytica* HM-1:IMSS (red) and all *E*. *histolytica* clinical isolates reported in yellow (369), dark green (373), violet (1238), pink (8026) and dark blue (3291); “Spectra view” of the 2 discriminating peaks of *E*. *histolytica* HM-1:IMSS average spectra (red) compared to all *E*. *histolytica* clinical isolates average spectra reported in yellow (369), dark green (373), violet (1238), pink (8026) and dark blue (3291) and *E*. *dispar* SAW760 average spectra (green) **(B)** “PCA-3D dot plot windows” showing the spectra of *E*. *histolytica* HM-1:IMSS (red) and *E*. *moshkovskii* Laredo (blue) clustering separately from the group including *E*. *dispar* SAW760 (green) and all *E*. *dispar* clinical isolates reported in yellow (1557), dark green (217), violet (2550), pink (1110), dark blue (240), brown (417), orange (368), and light blue (1382); “Spectra view” of the 3 discriminating peaks of *E*. *dispar* SAW760 average spectra (green) compared to all *E*. *dispar* clinical isolates average spectra reported in yellow (1557), dark green (217), violet (2550), pink (1110), dark blue (240), brown (417), orange (368), and light blue (1382) and *E*. *histolytica* HM-1:IMSS average spectra (red). **(C)** “PCA-3D dot plot windows” showing the spectra of the clinical isolate *E*. *histolytica* 1656 (yellow) clustering separately from *E*. *histolytica* HM-1:IMSS (red), *E*. *dispar* SAW760 (green) and *E*. *moshkovskii* Laredo, (blue); “Spectra view” of the discriminating peaks found for the clinical isolate *E*. *histolytica* 1656 average spectra compared to three reference strains average spectra.

Replicate spectra for each *E*. *histolytica* and *E*. *dispar* clinical isolate analyzed were loaded into ClinProTools software and the “PCA-3D dot plot window” was created (Fig. 4A and 4B, respectively). The “PCA-3D plot” obtained for *E*. *histolytica* clinical isolates showed that these strains cluster near *E*. *histolytica* HM-1:IMSS and were totally separate from *E*. *dispar* SAW760 and *E*. *moshkovskii* Laredo. Similarly, the “PCA-3D plot” obtained for *E*. *dispar* clinical isolates showed that these strains clustered near *E*. *dispar* SAW760 and were totally separate from *E*. *histolytica* HM-1:IMSS and *E*. *moshkovskii* Laredo.

The average spectra created for each clinical isolate were compared with the average spectra of the *E*. *histolytica* and *E*. *dispar* reference strains to verify the presence or the absence of the species-specific discriminating peaks (“Spectra view” Fig. 4A and 4B, respectively, and Table 1). All clinical isolates except *E*. *histolytica* strain 1656 showed the discriminating peaks expected for the respective species. This strain displayed 2 of the 3 discriminating peaks of *E*. *dispar* (4,714 and 5,541 Da) and no specific peaks of *E*. *histolytica* were found (“Spectra view” Fig. 4C and Table 1).

The “PCA-3D plot” showed that strain 1656 clustered separately both from *E*. *histolytica*, *E*. *dispar*, and *E*. *moshkovskii* reference stains (“PCA-3D dot plot window” Fig. 4C).

The sequences of *E*. *histolytica* 1656, *E*. *histolytica* 8026, and *E*. *histolytica* HM-1:IMSS 18S-rDNA fully matched *E*. *histolytica* GenBank X56991 and the sequences of *E*. *dispar* 1557 and *E*. *dispar* SAW760 fully matched *E*. *dispar* GenBank Z49256.

**Fig 5:**
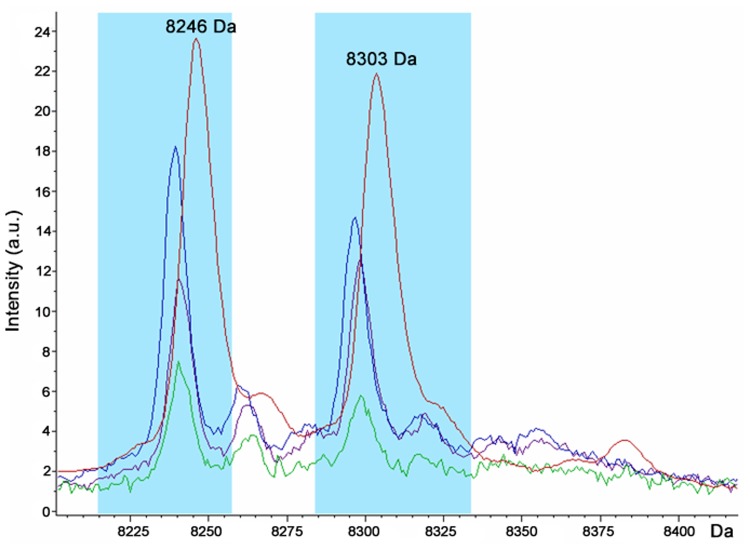
Spectra view of the discriminating peaks found for *E*. *histolytica* 373 average spectra obtained after 12h (green), 24h (violet), and 48h (blue) inoculation of the original fecal sample in Robinson’s medium compared to *E*. *histolytica* HM-1:IMSS average spectra (red).

The spectra obtained by MALDI-TOF MS from the fecal sample 373 at different times of incubation, showed the presence of the *E*. *histolytica* specific peaks after 12h of incubation (Fig. 5). The discriminating peaks of *E*. *dispar* were also detected in the fecal sample 1382 after 24h of incubation.

A minimum concentration of 10^6^ trophozoites/g of feces (detection limit) was required to observe the discriminating peaks of *E*. *histolytica* and *E*. *dispar* by MALDI-TOF MS directly on experimentally seeded fecal specimens.

## Discussion

For twenty years, mass spectrometry has been used as a particularly powerful tool for analysis and characterization of proteins. However, it is only recently that this technology, especially MALDI-TOF MS, has entered the field of routine clinical microbiology. MALDI-TOF MS allows easier and faster diagnosis of human pathogens than conventional phenotypic and molecular identification methods, with unquestionable reliability and cost–effectiveness [3,18].

However, in human parasitology mass spectrometry has had limited application, such as the detection of malarial hemozoin in blood, using technologies different from MALDI-TOF [19], or parasite identification or detection of their biomarkers by MALDI-TOF MS (eg. [1,3,5–7,20]).

In this study, MALDI-TOF MS was applied for the first time to the identification and differentiation of *E*. *histolytica* and *E*. *dispar* strains isolated from clinical samples.

Amebiasis, the parasitic infection caused by *E*. *histolytica*, is one of the most common parasitic infections worldwide. While it is still an important public health problem in developing countries, rapid differential diagnosis of amebiasis is crucial in terms of appropriate treatment choices in developed countries also [9,11]. For this reason, more rapid and specific methods are needed in order to differentiate the non pathogenic *E*. *dispar* from *E*. *histolytica* as they are indistinguishable in their cyst and trophozoite forms, except in cases of invasive disease when *E*. *histolytica* trophozoites may contain ingested red blood cells [21]. *E*. *moshkovskii*, which is morphologically indistinguishable from *E*. *histolytica* and *E*. *dispar*, has been considered to be primarily a free-living ameba, but it has occasionally been shown to infect humans [22]. It was included in this study to confirm the specificity of the proteic peaks proposed for *E*. *histolytica*/*E*. *dispar* differentiation. For the same reason one human isolate of *Entamoeba coli* was tested and the specific peaks of *E*. *histolytica* and *E*. *dispar* were not found (data not shown). The accurate differential diagnosis of intestinal infection with *E*. *histolytica* and *E*. *dispar* is important for two reasons: first to understand the worldwide distribution of the species individually, and second, to prevent unnecessary chemotherapy in patients infected with *E*. *dispar* [9,11,17].

In spite of nearly 20 years of clinical evidence showing molecular methods to be significantly faster and often more accurate with respect to diagnosis, many laboratories, particularly those performing diagnostic parasitology, are yet to adopt them as part of their routine practice [23].

Amplification of ameba DNA fragments by PCR has proven to be a sensitive and specific method for the diagnosis of amebiasis, circumventing the insensitivity of microscopic or culture-based diagnosis [9,21,24]. However, this molecular method remains cumbersome and expensive, as well as not being widely performed in parasitology laboratories. Clearly, the development and validation of alternative, rapid and universal identification methods are warranted; MALDI-TOF MS methods may fill some of these critical gaps [23].

MALDI-TOF MS is being used increasingly for clinical microbiological diagnosis although it is poorly described in diagnostic parasitology and it could be used in particularly in laboratories located in non-endemic areas.

A previous study [25] used a different mass spectrometry technique (Surface-enhanced laser desorption ionization-Time of Flight—SELDI-TOF MS- Protein Chip assay) to analyze different isolates of *E*. *histolytica* and *E*. *dispar*, but it wasn’t able to distinguish the two species on the basis of the peaks pattern and didn’t report the significance of the revealed peaks.

Our study demonstrated that MALDI-TOF MS can be used in a non-endemic setting for differentiating *E*. *histolytica* from *E*. *dispar* directly in xenic cultures based on the presence/absence of 5 specific peaks. Two discriminating peaks for *E*. *histolytica* and 3 peaks for *E*. *dispar* grown in axenic culture were found. These peaks did not show interference with the peaks found for the 3 different culture media used in this study, nor with those of *E*. *moshkovskii* Laredo. For this reason these 5 discriminating peaks may be considered good markers to differentiate *E*. *histolytica* from *E*. *dispar*. In addition, it was also possible to identify 3 differentiating peaks for *E*. *moshkovskii*.

In our hands, the 5 discriminating peaks allowed us to differentiate 13 of the 14 *Entamoeba* spp. clinical isolates grown in Robinson’s medium (8 *E*. *dispar* and 5 *E*. *histolytica*); the results matched those obtained by Real-time PCR. For one clinical isolate (strain 1656) identification by MALDI-TOF MS was not possible, although it had been identified as *E*. *histolytica* by Real-time PCR and sequencing. Moreover, this strain was isolated from a patient with dysentery and positive for *E*. *histolytica* antibodies. It is noteworthy that this strain clustered separately from the *E*. *histolytica*, *E*. *dispar*, and *E*. *moshkovskii* reference strains. In the light of this result, we hypothesize the presence of amino acid/posttranslational differences in the proteins detected by MALDI-TOF MS for this strain. Further studies would be needed to verify this. However, when using MALDI-TOF MS as first-line diagnostic assay the identification of a similar strain, if any, in the future should be confirmed by Real-time PCR.

The data obtained for the fecal samples 373 and 1382 were interesting. Although the discriminating peaks for *E*. *histolytica* (8,246 and 8,303 Da) and for *E*. *dispar* (4,714 Da; 5,541 Da; 8,207 Da) were not found directly in the fecal sample, these peaks were detected in the corresponding fecal sample after only 12h of incubation for *E*. *histolytica* and 24h for *E*. *dispar* in Robinson’s medium without serum, that is the first step before the transfer of an aliquot in the complete medium when the culture for protozoa is performed. This result underlines the reliability of the discriminating peaks found in this study: they were detected also in the presence of fecal material, which makes analysis by mass spectrometry difficult [1]. As expected, the ability to detect discriminating peaks directly on fecal specimens by MALDI-TOF MS is limited by the need for a high concentration of trophozoites/g of feces. Our results confirm reports in the literature regarding the identification of bacteria and fungi by MALDI-TOF MS, which required a microbial growth in pure culture (liquid or solid) [23].

Compared to molecular methods such as Real-time PCR assays (detection limit 10 trophozoites/g of feces [9]), the main advantages of MALDI-TOF MS are that it is far less labour intensive, less expensive (excluding the cost of the instruments), and it does not require multiple physically separated areas. However, although MALDI-TOF MS required a minimum concentration of 10^6^ trophozoites/g of feces, our study showed that this could be a new approach to discriminate between *E*. *histolytica* and *E*. *dispar* using *in vitro* xenic cultures; in our hands, it is also potentially promising for the rapid detection of these species in fecal samples after a short step in Robinson’s medium without serum. This study is the first describing this procedure in a laboratory of diagnostic parasitology located in a non-endemic area. The increasing use of MALDI-TOF MS in clinical microbiology laboratories could lead to its application to other aspects of diagnostic parasitology, and this could be the goal of a further work.

## Materials and Methods

### Design of the study and Ethical Statement

In this study, 3 reference amebic strains *E*. *histolytica* HM-1:IMSS, *E*. *dispar* SAW760, and *E*. *moshkovskii* Laredo (kindly provided by Dr. Graham Clark, Faculty of Infectious and Tropical Diseases, London School of Hygiene and Tropical Medicine, UK) were used to verify the presence of specific proteic peaks of these species by MALDI-TOF MS analysis. The *E*. *histolytica* HM-1:IMSS and *E*. *moshkovskii* Laredo strains derived from ATCC collection. *E*. *dispar* SAW760 strain was axenized directly by Dr. Graham Clark [26]. Genomic sequences of these strains were reported [17]. In order to verify the reliability of the discriminating peaks found by mass spectrometry analysis, different strains belonging to the species *E*. *histolytica* (8026, 369, 373, 1238, 1656, 3291) and *E*. *dispar* (1557, 217, 240, 417, 368, 1110, 2550, 1382) isolated from 14 fecal samples in our laboratory and identified by a 18S rDNA Real-time PCR, as previously described [9], were analyzed by MALDI-TOF MS. These strains currently belong to our collection and they are used in our laboratory for research [9,11].

Two fecal samples from which 2 strains (*E*. *histolytica* 373 and *E*. *dispar* 1382) out of the 14 included in this study were isolated were also analyzed by MALDI-TOF MS using the same procedure described below.

For strains 8026, 1557, and 1656 the amplicons obtained by Real-time PCR were also sequenced as previously described [9,11], in order to confirm species identification. The Real-time PCR assay was also performed for the reference strains used and the amplicons obtained were sequenced, as previously described [9].

The samples analyzed in this and previous studies had been obtained by the University Hospital of Parma for routine diagnosis purposes, and no approval by the Institutional Review Board was required because of the laboratory diagnosis results had been reported in the medical records of the patients as a diagnostic answer to a clinical suspicion of amebiasis. Ethical approval at the University Hospital of Parma is required only in cases where the clinical samples are to be used for applications other than diagnosis. According to Italian Ministry of Health guidelines on amebiasis laboratory diagnosis, only verbal informed consent of the patients is requested on hospital admission. At the University Hospital of Parma informed consent procedures for the laboratory diagnosis of infectious diseases other than HIV serology do not require local committee approval because they are included in the Italian Public Health Legislation. The verbal informed consent from patients was not required for this kind of investigations (diagnosis of intestinal parasitosis) at the University Hospital of Parma because a clinical report was produced for each sample arrived with a medical order. In order to document the process, physicians must write a medical order with the personal and clinical data of each patient who asks for health assistance in a public hospital including the University Hospital of Parma, without any interaction with the patients by the Authors. Fecal samples were analyzed anonymously for all the assays used. The anonymization of the samples was performed by the technical staff of the laboratory and Authors had no access to the data prior their anonymization.

### Cultivation of amebic strains and treatment of fecal sample

The reference strains *E*. *histolytica* HM-1:IMSS and *E*. *dispar* SAW760 were cultivated in LYI-S-2 axenic medium as previously described [27] at 37°C for 72 h, while *E*. *moshkovskii* Laredo was maintained in the same medium at room temperature for 15 days.

All the 14 field isolates were cultivated in monoxenic cultures at 37°C in Robinson’s medium (containing *Escherichia coli* ATCC 8739) in 5% CO_2_ enriched atmosphere for 48/72h as previously described [9,27].

Two fecal samples (373 and 1382) were inoculated in Robinson’s medium without serum (BR) [27] and incubated for 1, 3, 6, 9, 12, 24, 48h and 9, 12, 24, 48h, respectively.

### Sample preparation for MALDI-TOF MS analysis

Cultures at a concentration of approximately 1x10^6^ trophozoites/ml were subjected to protein extraction in ethanol/formic acid as previously described [28,29] with some modifications. Briefly, a 1 ml aliquot of each culture was centrifuged at 3,000 x *g* for 10 minutes and the supernatant was discarded. The pellet obtained was subjected to two subsequent washing steps, first in 1 ml of PBS and then in 1 ml of distilled water both followed by centrifugation at 3,000 x *g* for 10 minutes. The pellet obtained was suspended in 300 μl of distilled water and then 900 μl of absolute ethanol were added. The subsequent extraction stages were performed as previously described [28–30]. Each time samples were processed, LYI-S-2 axenic medium and/or Robinson’s medium with and without the addition of *Escherichia coli*, were subjected to the same protein extraction procedure and used as a control. In each experiment, the “Bacterial Test Standard” (Bruker Daltonics, Germany) for calibration was used according to the manufacturer’s instructions.

In order to investigate the usefulness of MALDI-TOF MS for the detection of *Entamoeba* sp. discriminating peaks directly in fecal samples, strains *E*. *histolytica* 8026 and *E*. *dispar* 2550 were used to prepare fecal samples experimentally seeded with amebic cells. The fecal samples used were negative by both Real-time PCR for the presence of *E*. *histolytica* and *E*. *dispar*, and culture for protozoa. Serial ten-fold dilutions (from 10^6^ to 10^4^) of cells from *E*. *histolytica* and *E*. *dispar* cultures (1ml) were mixed with an equal volume of sterile culture medium containing 1 g of human feces. One ml of each dilution was centrifuged at 3,000 x *g* for 10 minutes and the pellet obtained was subjected to the same protein extraction procedure described above.

The two fecal samples (373 and 1382) from which the strains *E*. *histolytica* 373 and *E*. *dispar* 1382 were isolated, were also analyzed by MALDI-TOF MS: the sample 373 after 1, 3, 6, 9, 12, 24, and 48h of incubation in BR medium and the sample 1382 after 9, 12, 24, and 48h of incubation in the same medium. One ml of each culture containing fecal material collected at the times described was centrifuged at 3,000 x *g* for 10 minutes and the pellet obtained was subjected to the same protein extraction procedure described above.

### MALDI-TOF MS: spectra acquisition

Proteic extracts were analyzed by MicroFlex LT mass spectrometer (Bruker Daltonics, supplied by Becton Dickinson, Italy); spectra were acquired using MBT_Standard method (positive linear mode; laser frequency 60 Hz; ion source voltage 20 kV; mass range 2-20kDa) in manual mode acquisition with overall 520 laser-shot, instead of the 240 set in the MBT_Standard method, in order to obtain a clear signal, by 40 shot steps. Each shot step was made in different points of the well with the laser intensity variable in a range of 30–50% for each single shot step.

For each reference strain and clinical isolate at least 4 independent experiments using 4 independent cultures on 4 different days were run (inter-assay reproducibility) and 20 replicates/run were analyzed in order to ensure the reproducibility of the results obtained (intra-assay reproducibility).

In order to minimize the variability associated with technical or biological parameters, the experiments were performed under controlled cultivation and sample preparation conditions and consistent technical configurations, assuring a high repeatability and reproducibility between experiments.

### Spectra analysis

For all the spectra obtained by MALDI-TOF MS manual acquisition “Smoothing” and”Baseline” were performed using Flex Analysis software (version 3.3 Bruker Daltonics). The replicates with an intensity <10^4^ arbitrary units as well as those with a profile significantly different from the others were eliminated. In order to select the peaks discriminating between *E*. *histolytica* HM-1:IMSS and *E*. *dispar* SAW760 at least 10 replicates for each strain were chosen and imported into ClinProTools statistical software (version 2.2, Bruker Daltonics).

To assess the specificity of the discriminating peaks the same software was used to analyze the spectra obtained for *E*. *moshkovskii*.

In order to compare the strains, an equal number of spectra per strain were required: 10 spectra obtained for each of the 17 strains used in the study (3 reference strains and 14 field isolates) and 10 spectra obtained for each medium (LYI-S-2 and Robinson’s with and without *E*. *coli*) were loaded into the program and automatically recalibrated. Statistical testing of the datasets was performed on the basis of Principal Component Analysis (PCA) and the results were displayed in a three-dimensional score plot generated by the software. PCA reduces the variability of the complex datasets, automatically generating a set of new variables called the Principal Component (PC). The statistical tool PCA was applied to the datasets analyzed to visualize the homogeneity and heterogeneity of the proteic spectra.

The presence/absence of each discriminating peak, found by software on the basis of different statistical parameters, was evaluated by comparison of each average spectrum automatically created from the replicates of each strain and from the three different media used. A Receiver Operating Characteristic (ROC) curve for each peak was calculated; this gives a graphical overview of the specificity and sensitivity of a test, and in this case an evaluation of the discriminating quality of a peak. The ROC curve view takes into account only one peak as a test criterion and indicates this peak by an Area Under Curve (AUC) value. An AUC value of 0 indicates that the peak under consideration is not discriminating while an AUC value of 1 indicates that the peak is discriminating.

The significance of the discriminating peaks with their characteristic molecular weights was evaluated by the Analysis of Variance Test (ANOVA): the p-value obtained provides a measure of the probability of the strength of an association/dissociation among the different specific peaks for the species analyzed. Differences were considered significant when p <0.05.

### SDS-PAGE and protein extraction from polyacrylamide gel

The method used for sodium dodecyl sulfate polyacrylamide gel electrophoresis (SDS-PAGE) and protein extraction was the same previously described [31].

Briefly, amebic cultures at a concentration of 0.5x10^6^ trophozoites/ml were subjected to protein extraction in ethanol/formic acid after freezing at -20°C and centrifugation at 100,000 x *g* for 1h at 4°C. The pellet was treated with 50 μl Laemmli buffer (50 mM Tris-HCl pH 6.8; 100 mM dithiothreitol; 25% (w/v) glycerol; 2% SDS; 0.1% bromophenol blue) and heated at 95°C for 5 minutes. Subsequently 20 μl of the solution obtained were analyzed by SDS-PAGE with the following concentration conditions: 4% acrylamide/N,N bisacrylamide for the stacking gel and 20% for the running gel. A low range protein standard (Sigma-Aldrich, Milan, Italy) (MW 6,500–66,000) and the Polypeptide SDS-PAGE Molecular Weight Standards (Bio-Rad, Milan, Italy) (MW 1,423–26,625) were used as the molecular weight marker. The run was done at 50V for 40 min., and at 70V till the end (about 4h). Coomassie brilliant blue (Bio-Rad) gel staining was used.

Bands of interest were excised for protein extraction and placed in 1.5 ml-colourless tubes. After washing with 300 μl of high purity liquid chromatography (HPLC) grade water, each excised gel band was vortexed for 10 minutes in 100 μl 10% acetic acid. The solution was removed and the gel piece washed thoroughly with 300 μl HPLC grade water, then with 100 μl of acetonitrile, followed by HPLC grade water, and finally methanol for 20 minutes. To completely de-stain the gel, each gel piece was dipped into 100 μl of formic acid:HPLC grade water:isopropanol (1:3:2, v/v/v) solution and vortexed until the gel piece became colourless. Finally, each gel piece was partially dried, crushed in small pieces, and the proteins were extracted by adding 20 μl of alpha-cyano-4-hydroxycinnamic acid (HCCA). Molecular weight marker bands in the range of interest (3,496 to 14,437 Da) were also excised and subjected to the same procedure.
